# Exploration of Vaccination Attitudes Among Parents and Caregivers in a Rural Appalachian Health Clinic

**DOI:** 10.13023/jah.0402.07

**Published:** 2022-07-01

**Authors:** Radwa Omar, Karen Hande, Natasha McClure

**Affiliations:** Vanderbilt. University School of Nursing, radwaallen@outlook.com; Vanderbilt University School of Nursing; Vanderbilt University School of Nursing

**Keywords:** Appalachia, childhood vaccine, vaccine attitude, Appalachian health

## Abstract

At a rural Appalachian health clinic in Kentucky, 20% of patients under 18 years were not up to date with the CDC-recommended immunization schedule. Reasons parents or caregivers chose to delay or refuse their child’s immunizations were explored using the Caregiver Vaccination Attitude Scale. High levels of trust in the healthcare provider and self-reported vaccine knowledge highlight opportunities for rural healthcare providers to apply evidence-based communication strategies to address vaccine hesitancy and promote the safety and health of the entire community.

## INTRODUCTION

Parental refusal or delay of childhood immunizations has raised significant concern on a national level over the past decade.^1^ Delayed or refused immunization has been linked to an increased risk of preventable diseases in pediatric patients.^1^ Communities with unimmunized children have experienced small, but recognizable, growth in the prevalence of these previously eradicated communicable diseases.^2^ These communities are at risk for disease outbreak, which may have local, national, and international effects.^2^

Parents’ reported lack of trust in their healthcare provider influences their decision not to vaccinate.^3^ Trust is paramount and perhaps more important than individual attitudes with regard to vaccine behavior.^4^ Larger, systemic reviews underscore the importance of respect and empathy toward parental concerns related to vaccines^5^ and have linked insufficient discussions (both in terms of length and depth) and dismissiveness of concerns with lower vaccine uptake.^6^

## A NOTE ON VACCINATION ATTITUDES

A chart review of patients who visited a primary care clinic in rural Kentucky during 2020 determined that approximately 20% (n=1,500) of their pediatric patients were not in compliance with the current laws concerning school attendance and immunization schedule and guidelines.^7^–^9^ To increase vaccination rates in this rural community-based health clinic, attitudes toward immunizations were assessed. Parents or caregivers who had delayed or refused to vaccinate their children within the past 12 months were asked to complete the Caregiver Vaccination Attitude Scale (CVAS) to measure (1) beliefs about the effects of vaccines, (2) knowledge regarding vaccine preventable diseases, (3) knowledge about available locations to receive immunizations, and (4) trust in healthcare providers.^10^

Seventy-five percent of the respondents reported delaying a vaccination, despite high levels of trust in their healthcare provider (70%), and knowledge about where (95%) and when (85%) to receive vaccines. A large number of those surveyed (80%) reportedly felt they should be able to selectively choose the vaccines they believe their child needs and that it is better to develop immunity by getting sick than through vaccination (45%).

Seventy percent agreed that they trust the care and information offered by nurses and nurse practitioners (NPs), likely indicating this is not the most important factor influencing vaccine delay and refusal among this clinic population. Data highlighted that parents/caregivers did not agree with the prospect of multiple vaccines administered in a single setting and find the overall number of vaccines to exceed what they believe is necessary. Eighty percent of parents agreed that people in their rural Appalachian community have expressed concerns that a child might have a serious side effect from a vaccination.

## IMPLICATIONS

Understanding benefits and perceived barriers for patients in a rural Appalachian health clinic may enable providers to effectively impact vaccination by cueing action through personalized approaches to education and decision-making support. The effectiveness of any vaccine communication approach assumes the patient considers their healthcare provider a trusted source of information. The parents/caregivers at this clinic reported high levels of trust in their healthcare provider and self-reported vaccine knowledge, but the corresponding low uptake of vaccines may indicate the need for a more strategic response to delay and refusal based on information from this survey.

The presumptive and participatory approaches are frequently used by clinicians in vaccine communication.^11^ The presumptive approach often takes the form of a simple recommendation statement at the end of a well-child visit when the provider informs parents about immunizations the child should receive as part of the visit.^11^ This approach was noted to have higher rates of acceptance of vaccines, but lower rates of parent satisfaction with the visit.^12^ The participatory approach engages the parent in discussion, asking their thoughts about vaccines. This was found to have lower rates of vaccine uptake but correspondingly high rates of parent satisfaction with the visit.^12^

Nurses and NPs in rural healthcare settings can build on existing trust and attempt to increase compliance with vaccine recommendations in the long term by acknowledging the underlying power dynamics of the patient–provider relationship, strengthening rapport through dialogue, and showing concern and empathy via answering questions.^4^ Leveraging parents’ confidence in clinic NPs, engaging parents in discussions about their beliefs and concerns, and addressing knowledge gaps all serve as crucial steps for maintaining positive relationships and potentially improving vaccination rates among this population of patients.
